# *In Silico* Functional Pathway Annotation of 86 Established Prostate Cancer Risk Variants

**DOI:** 10.1371/journal.pone.0117873

**Published:** 2015-02-06

**Authors:** Lenora W. M. Loo, Aaron Y. W. Fong, Iona Cheng, Loïc Le Marchand

**Affiliations:** 1 Cancer Epidemiology Program, University of Hawaii Cancer Center, Honolulu, Hawaii, United States of America; 2 Epidemiology Research Department, Cancer Prevention Institute of California, Fremont, California, United States of America; UC Davis Comprehensive Cancer Center, UNITED STATES

## Abstract

Heritability is one of the strongest risk factors of prostate cancer, emphasizing the importance of the genetic contribution towards prostate cancer risk. To date, 86 established prostate cancer risk variants have been identified by genome-wide association studies (GWAS). To determine if these risk variants are located near genes that interact together in biological networks or pathways contributing to prostate cancer initiation or progression, we generated gene sets based on proximity to the 86 prostate cancer risk variants. We took two approaches to generate gene lists. The first strategy included all immediate flanking genes, up- and downstream of the risk variant, regardless of distance from the index variant, and the second strategy included genes closest to the index GWAS marker and to variants in high LD (r^2^ ≥0.8 in Europeans) with the index variant, within a 100 kb window up- and downstream. Pathway mapping of the two gene sets supported the importance of the androgen receptor-mediated signaling in prostate cancer biology. In addition, the hedgehog and Wnt/β-catenin signaling pathways were identified in pathway mapping for the flanking gene set. We also used the HaploReg resource to examine the 86 risk loci and variants high LD (r^2^ ≥0.8) for functional elements. We found that there was a 12.8 fold (p = 2.9 x 10^-4^) enrichment for enhancer motifs in a stem cell line and a 4.4 fold (p = 1.1 x 10^-3^) enrichment of DNase hypersensitivity in a prostate adenocarcinoma cell line, indicating that the risk and correlated variants are enriched for transcriptional regulatory motifs. Our pathway-based functional annotation of the prostate cancer risk variants highlights the potential regulatory function that GWAS risk markers, and their highly correlated variants, exert on genes. Our study also shows that these genes may function cooperatively in key signaling pathways in prostate cancer biology.

## Introduction

Genome-wide association studies (GWAS) have identified hundreds of genetic variants associated with cancer [1] [2]; yet, most risk alleles are associated with a modest disease risk (OR<1.5). Moreover, additional susceptibility variants will be identified, with growing sample sizes and the application of high-throughput sequencing technologies. The important next steps involve fine mapping of association signals followed by functional characterization of the putative causal variants. In this era of extensive characterization of the human genome with the International HapMap and 1000 Genomes project, data curation of genomic modifications involved in gene regulation by the Encyclopedia of DNA Elements (ENCODE), and the high-resolution molecular characterization of common cancers by The Cancer Genome Atlas (TCGA), we can attempt to integrate this information to characterize the biological mechanisms that are impacted by cancer risk variants.

Prostate cancer will affect one in seven men in their lifetime and is the second leading cause of male cancer-related deaths in the U.S. It is a heterogeneous disease with variable clinical course. Although most prostate tumors are indolent, some are aggressive, spreading to the bladder, rectum, and bone. Family history is an established risk factor for prostate cancer, supporting the observation that there is a strong genetic component to the disease [3–5]. Men with a family history of prostate cancer are over 3 times more likely to develop prostate cancer, and their risk increases with two or more affected first-degree relatives [6]. Twin studies demonstrate that the contribution of heritable factors is as high as 42% for developing prostate cancer [4].

The majority of the 86 prostate cancer risk variants that have been identified to date are located in non-coding intronic or intergenic regions [2]. Therefore, annotating the functional elements that are associated with these risk variants, correlated variants in high linkage disequilibrium along with neighboring genes and functional networks, may help to improve our understanding of the biological mechanisms involved in the etiology of prostate cancer.

## Methods

### Prostate cancer risk alleles

Eighty-six prostate cancer risk variants were abstracted from the National Human Genome Research Institute (NHGRI) GWAS catalogue (as of April 2013) [1,2] and the International Collaborative Oncological Gene-environment Study (iCOGs) consortium [7] that met the genome-wide significance level of p ≤ 5 x 10^–8^ (S1 Table).

### Prostate cancer risk allele associated gene lists

We took two approaches to generate gene lists to map biological pathways potentially underlying the genetic signals represented by the prostate cancer risk alleles. With the first approach, we included all immediate flanking genes (closest gene on either side of the risk allele), up- and downstream of the risk allele, regardless of distance from the index variant. With the second approach, we analyzed all genes closest to the index marker and all other variants in high LD (r^2^ ≥0.8 in Europeans from the 1000 Genomes Project) with the index marker, within a 100 kb window up- and downstream of the SNP, considering most enhancer elements are located within 100 kb of their target genes.

### HaploReg analysis

A total of 86 SNPs were used for the functional annotation analysis of established prostate cancer risk alleles. The HaploReg v2 (http://compbio.mit.edu/HaploReg) resource and database was used to identify biofeatures in sequences containing the prostate cancer risk variants and SNPs in in high LD (r^2^ ≥0.8 in Europeans from the 1000 Genomes Project) [9]. Functional elements located in the same regions as index and correlated SNPs were identified in ENCODE [8]. The HaploReg tool identified evolutionarily conserved regions based on SiPhy (SIte-specific PHYlogenetic) analysis [10]. Variants were annotated with potential effects on regulatory motifs based on existing databases such as TRANSFAC, JASPAR, and PBM [9].

### Protein Motif Prediction

PolyPhen and PROVEAN analysis was applied to coding SNPs to predict the structural and functional effects of amino acid substitutions [11,12].

### Functional Network and Pathway Prediction

Ingenuity Pathway Analysis (IPA; http://www.ingenuity.com/) was used to identify potential functional networks and pathways. IPA Core Analysis was applied to the gene lists to identify direct and indirect interactions based on the IPA Knowledge Base, a repository of curated biological interactions and functional annotations based on existing literature.

### TCGA analysis of prostate cancer tumor tissue

The Cancer Genome Atlas (TCGA) database of prostate cancer tumor tissue gene expression profiles was queried using the cBioPortal for Cancer Genomics analysis resource (http://www.cbioportal.org/public-portal/) [13,14].

## Results

### Functional Profiles of Genes Flanking Risk SNPs

**Fig 1:**
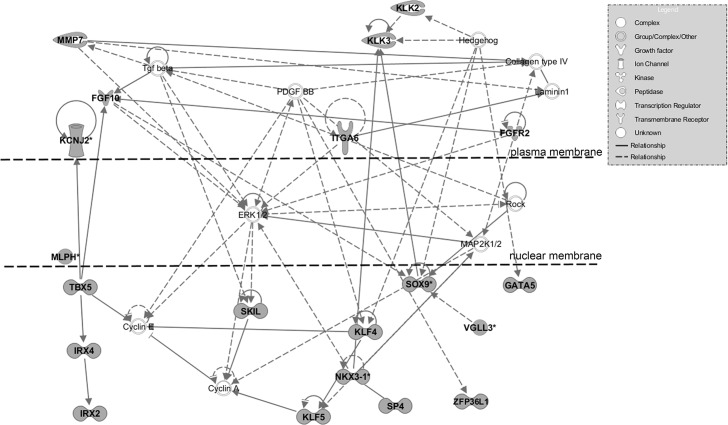
Gene-gene interactions among genes flanking prostate cancer risk alleles. A total of 97 unique genes contained or flanking the 86 prostate cancer risk loci. Gene-gene interactions were identified using the Ingenuity Pathway Analysis software. The most significant functional network demonstrating connectivity between genes was identified as having a potential function in *Organismal Development*, *Embryonic Development*, *and Organ Development*. The representative gene products are listed and putative functions listed in the legend. Gene products shaded in gray represent genes originating from the gene list.

A total of 97 annotated genes were identified to be the most immediate neighboring genes up- and downstream of the 86 prostate cancer risk variants (S2 Table). The IPA software was used to characterize the functional composition of the 97 genes. Of the 97 genes, 56 were identified to have a functional role in cancer, with enrichment for genes associated with prostate cancer that included: HNF1 homeobox B (*HNF1B)*, kallikrein-related peptidase 2 *(KLK2)*, kallikrein-related peptidase 3 *(KLK3)*, lemur tyrosine kinase 2 *(LMTK2)*, NK3 homeobox 1 *(NKX3-1)*, and solute carrier family 22 (organic cation transporter), member 3 (*SLC22A3)*. IPA also identified potential gene-gene interactions and networks which suggested that the gene list was composed of genes that could function collectively in specific biological mechanisms. The top functional network included 20 focus molecules from the gene list and has a role in *Organismal Development*, *Embryonic Development*, *and Organ Development* (Fig. 1). Other functional networks identified based on the 97 gene list, were *Cell-To-Cell Signaling and Interaction*, *Connective Tissue Disorders; Cell Death and Survival*, *Cancer*, *Organismal Injury and Abnormalities* (Table 1). These results support the observation that the genes flanking the prostate cancer risk alleles have the potential for functional connectivity to form biological networks that have a role in development, cellular signaling, cell death and survival, and cancer.

**Table 1:** Gene-gene interaction networks and associated diseases and biological functions among genes flanking prostate cancer risk variants.

Molecules in Network	Score	Focus Molecules	Top Diseases and Functions
Alp,calpain,Collagen type IV,Cyclin A,Cyclin E, E2f,ERK1/2, **FGF10,FGFR2,GATA5**,Hedgehog,**IRX2,IRX4,ITGA6, KCNJ2, KLF4,KLF5,KLK2,KLK3**,Laminin1,MAP2K1/2, **MLPH, MMP7, NKX3-1**, PDGF BB,Rb,Rock,**SKIL,SOX9,SP4,TBX5**,TCF,Tgf beta, **VGLL3,ZFP36L1**	42	20	Organismal Development, Embryonic Development, Organ Development
Alpha tubulin,**ASCL2**,CD3,Cg,Ck2,Creb,ERK,FSH,HISTONE, Histone h3,IL12 (complex), Immunoglobulin, Insulin,**KRT8,LILRB2**, Mapk,Mmp,**MMP20**, NFkB (complex), P38 MAPK,**PDLIM5**, Pkc(s),**PPP1R14A,RAD23B**,RNA polymerase II, **SLC25A37, STC2**,TCR,**TH,TRIM8**,trypsin, **TTLL1,TUBA1C,VAMP8**, Vegf	25	14	Organismal Development, Cell-To-Cell Signaling and Interaction, Connective Tissue Disorders
26s Proteasome,**AFM**,Akt,Ap1,**AR**,BCR (complex),** BIK**,caspase,** CLDN11**, Collagen type I, **CXXC4**,cytochrome C,estrogen receptor,** FERMT2**,Hdac,**HNF1B**,Hsp27,Hsp70,Hsp90,Igm,IL1,**MDM4**,Mek, **MSMB**, N-cor, **NOTCH4**,Rxr,**SEMA6D**,Sos,SRC (family),** TERT,TET2**, Ubiquitin,Wnt	24	13	Cell Death and Survival, Cancer, Organismal Injury and Abnormalities
APP,**ATP9B**,C18orf21,C4orf21,C9orf41,C9orf142,**CCHCR1**,CHAC2,CHID1,**CHMP2B**,CPT1A,DDHD2,EEPD1,FAM207A,**FAM57A,FARP2**,HDAC1,HINT3,**KLF12,KRT78**, OCIAD2,PASD1,PUS7L,RABL3,** RAD51B**,RNASE11, **SALL3,SLC22A1,SLC22A2**, SUMO2,TBCC,**TIMM23B**, UBC,**ZGPAT**,ZNF720	23	13	Cell-To-Cell Signaling and Interaction, Molecular Transport, Small Molecule Biochemistry
**BOD1**,C1orf27,C4orf32,DCAKD,DESI2,**EEFSEC**,ELAVL1,EWSR1, **FAM84B**,FOPNL,GDPGP1,GRB2,KIAA2013,**LILRA3,LMTK2**,LRRC37B, MARVELD2,**MYEOV,NUDT10**,OR6T1,RASSF1,**RBM19**,RNF212, **SHROOM2**, SLC18B1,SLC41A1,**SP8**,SPRY2,** THADA**, TMEM127, TMEM209,UBC,VSIG10,ZNF529,ZSWIM1	19	11	Cell Signaling, Cellular Assembly and Organization, DNA Replication, Recombination, and Repair
C1orf52,COMMD7,**DPF1,EBF2,EHBP1**,FASTKD1,FOS,**GGCX**,Gm5451,HDDC2,IL4,LRCH3,NFKB1,Olfr1508,**RFX6**,RGD1561590/Sap18,**RGS17**,RIPK3,**SLC22A3**,SLC2A6,Snx3,SPON2,TMED5,TMEM131,TSPYL4,UBC,**VPS53,WDR11**,YY1,**ZBTB38**, ZNF195,ZNF287,ZNF415,ZNF571,**ZNF652**	19	11	Antimicrobial Response, Lymphoid Tissue Structure and Development, Organ Morphology
Actin,Ca2+,Calmodulin,Calmodulin-Ca2+-CaMKII+Calmodulin-Ca2,Calmodulin-Camk4-Ca2+,Calmodulin-CaMKI-Ca2+,Calmodulin-CaMKII-Ca2+,Calmodulin-Camkk-Ca2+,**COL6A3**, DMD, **GRHL1**,Jnk,KCNN1, **KCNN3**,KNDC1,**MYC**, N-arachidonylglycine, Nca,noladin ether,**OPHN1, PHOSPHO1**,PI3K (complex), Pka,platelet activating factor phosphatidate, PPEF1,PPEF2,**PRPH**,Ras, SHP2-PI3K-GAB2,**SQRDL**, TOR2A,TPCN1,**TPCN2**,TRPM3,vitamin K1	14	9	Cell-To-Cell Signaling and Interaction, Nervous System Development and Function, Cell Signaling

* Genes indicated in bold are on the genes flanking prostate cancer risk variants.

When these 97 genes were queried for alterations to gene expression, copy number, and mutation profiles in The Cancer Genome Atlas (TCGA) prostate adenocarcinoma dataset (n = 236), we found that more than half of the genes (65 of 97 genes) were altered in at least 5% of the tumors in the dataset, including 8 genes that were altered in >10% of the tumors (S3 Table).

**Fig 2:**
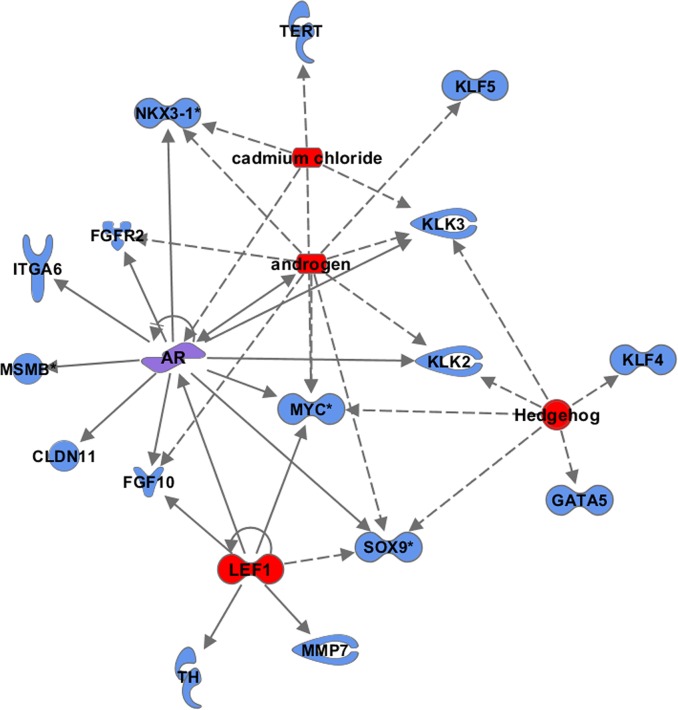
Upstream regulators among genes flanking prostate cancer risk alleles. The IPA Upstream Regulator tool was used to identify potential upstream regulators based on the statistical significance of genes in the gene list that function downstream of this regulator. The top 5 upstream regulators identified were androgen, androgen receptor (AR), lymphoid enhancer-binding factor 1 (LEF1), hedgehog (HH), and cadmium chloride. Upstream regulators (red); AR was both and upstream regulator and on the gene list (purple shading); genes from the gene list (blue).

The IPA Upstream Regulator tool was used to further identify critical signaling pathways that may functionally link together genes that may have a role in prostate cancer initiation and progression. This tool identifies potential upstream regulators based on the statistical significance of genes from the gene list that function downstream of the identified upstream regulator. Examples of upstream regulators include transcription factors, cytokines, microRNAs, receptors, kinases, chemicals and drugs. Of the 22 significant upstream regulators (Fisher exact test p< 1 x 10^–4^), identified by IPA, the five most significant upstream regulators were androgen, androgen receptor (*AR)*, lymphoid enhancer-binding factor 1 (*LEF1)*, hedgehog *(HH)*, and cadmium chloride (S4 Table). These upstream regulators point to the importance of genes that function in the androgen receptor, hedgehog, and Wnt/β-catenin signaling pathways in prostate cancer biology. Seventeen genes from the 97 gene list were identified to function downstream of these top 5 upstream regulators. Moreover, these results support the potential for crosstalk between these pathways in prostate cancer, as several of the downstream genes from the gene list shared the same upstream regulators (Fig. 2).

When we queried TCGA data with the 17 genes associated with the top five upstream regulators to determine if these genes were altered with regard to gene expression, copy number, and mutation in prostate adenocarcinoma (n = 236). We found that the majority of the genes from this subset (11 of 17) were altered in at least 5% of the 236 tumors. The most frequent altered gene was the tumor suppressor, *NKX3-1*, which was deleted or mutated in 30 of the 236 prostate cancers (12.7%) included in the TCGA dataset. Other upstream network genes that were altered in the TCGA tumors (in >5% of the 236) were v-myc avian myelocytomatosis viral oncogene homolog (*MYC)*, Kruppel-like factor 5 *(KLF5)*, integrin, alpha 6 *(ITGA6)*, microseminoprotein, beta *(MSMB)*, claudin 11 *(CLDN11)*, fibroblast growth factor receptor 2 *(FGFR2)*, SRY (sex determining region Y)-box 9 *(SOX9)*, fibroblast growth factor 10 *(FGF10)*, GATA binding protein 5 *(GATA5)*, and kallikrein-related peptidase 3 *(KLK3)* (S3 Table). Alterations to these genes in prostate tumors are consistent with the potential regulatory role of genetic variants to regulate genes involved in key signaling pathways in prostate cancer biology.

### Functional profiles of genes neighboring SNPs in high LD with prostate cancer risk alleles

**Fig 3:**
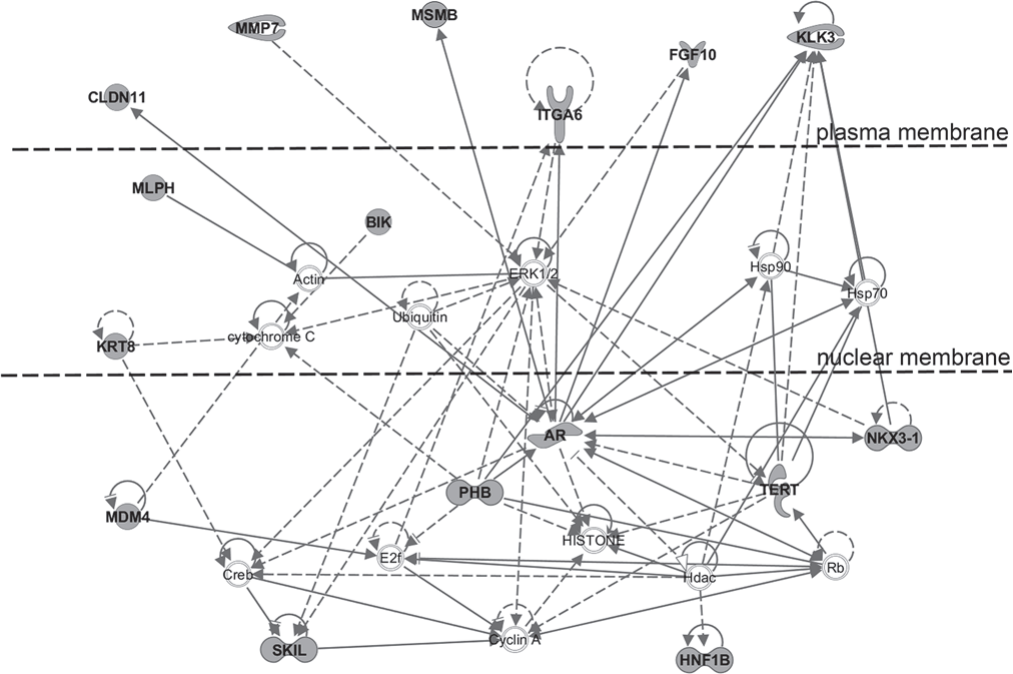
Gene-gene interactions among genes neighboring SNPs in high LD with prostate cancer risk alleles. A total of 78 unique genes contained or were located within 100 Kb of SNPs in high LD (r^2^>0.80). Gene-gene interactions were identified using the Ingenuity Pathway Analysis software. The most significant functional network demonstrating connectivity between genes was identified as having a potential function in *Cancer*, *Cellular Growth and Proliferation*, *and Organismal Injury and Abnormalities*. Gene products shaded in gray represent genes originating from the gene list.

A total of 1,594 individual SNPs (including the 86 index SNPs) were identified to be in high LD with the index SNPs and 81 focus molecules (78 annotated individual genes and 3 microRNAs) were within 100 kb of these SNPs (S5 Table). Network analysis, using IPA, to identify functional connectivity between the genes, indicated that the top functional network was *Cancer*, *Cellular Growth and Proliferation*, *and Organismal Injury and Abnormalities*, with 16 of the 78 genes from the gene list included in this network (Fig. 3). Additional associated network functions, such as *Hereditary Disorder; Organismal Injury and Abnormalities; DNA Replication*, *Recombination*, *and Repair; Cell Morphology; and Cellular Function and Maintenance*, were identified based on the 78 gene list and described in Table 2.

**Table 2:** Gene-gene interaction networks and associated diseases and biological functions among genes neighboring SNPs in high LD with prostate cancer risk variants.

Molecules in Network	Score	Focus Molecules	Top Diseases and Functions
26s Proteasome, Actin, **AR, BIK**, caspase, **CLDN11**, Creb, Cyclin A, cytochrome C, E2f,ERK1/2,estrogen receptor, **FGF10**,Hdac,HISTONE, **HNF1B**,Hsp70,Hsp90,Igm,** ITGA6,KLK3,KRT8**,Laminin1, **MDM4, MLPH,MMP7,MSMB,NKX3-1,PHB**, Rb,**SKIL**,TCF,**TERT**, trypsin,Ubiquitin	32	16	Cancer, Cellular Growth and Proliferation, Organismal Injury and Abnormalities
C10orf12,CCDC91,CERS5,CFDP1,DNAJB3,**EEFSEC,EHBP1**,EPT1,EWSR1,** FARP2,GGCX,IRX4**,KDM5D,LEPREL4,**LILRA3,LMTK2**,MAPK1IP1L, **MYEOV**,NBPF3,NLE1,NSUN4,PRTFDC1,RAB37,**RGS17**,SLC2A4,**SLC2A4RG, THADA**,TPGS2,UBC,**VAMP5**,VPS52,**VPS53**,ZBTB1,**ZBTB38**,ZNF184	28	14	Cancer, Hereditary Disorder, Organismal Injury and Abnormalities
ABL1,APOL5,**ARFRP1**,ASB16,CLDND1,D-glucose,**EBF2**,EMC9,EVL, FAM53C,G6PC2,GNB1,**GNGT2**,HNF4A,**MAT2A**,MTA2,NMNAT2,**NUDT11**,**NXPH3**, Pld,**RAD51B**, RBBP4,**RUVBL1,SALL3,SHROOM2,SLC22A1, SLC22A3**,SLC33A1,SLC35B1,SRC,TMEM128,TULP4,XPR1,YY1,**ZGPAT**	25	13	DNA Replication, Recombination, and Repair, Cell Morphology, Cellular Function and Maintenance
**ABI3,AFM**,Akt,Ap1,Calmodulin,Cg,Collagen type I,ERK,F Actin,**FERMT2**, FSH,Histone h3,Jnk,**KCNN3,KLF5,LIME1**,Mapk,NFkB (complex),** NGFR, NOTCH4**, P38 MAPK,**PDK1**,PI3K (complex), **PIK3C2B**,Pkc(s),**PPP1R14A**, Ras, RNA polymerase II,SRC (family),TCR,Tgf beta,**TRIM8**,Trk Receptor, **VAMP8**,Vegf	25	13	Cardiovascular System Development and Function, Cell Morphology, Cellular Development
ANGPTL7,ANXA2,APP,ATL3,**B4GALNT2**,C12orf5,CALML4,**CCHCR1, COL6A3**, DCDC2,**GRHL1**,LETM2,**LRRN2**,MAB21L2,mir-584,MPV17L, OARD1,**PDLIM5**, PRPF40B,**RAD23B, RFX6,SESN1**, SNRPB,STAT3,**TET2**,TGFB1,THBS1,TP53, **TUBA1C**,UBL3, VAT1L,VCL,**VGLL3**,VWF,**ZNF652**	23	13	Cell Cycle, Cancer, Cell Morphology

* Genes indicated in bold are on the genes neighboring SNPs in high LD (r^2^>0.80) prostate cancer risk variants.

**Fig 4:**
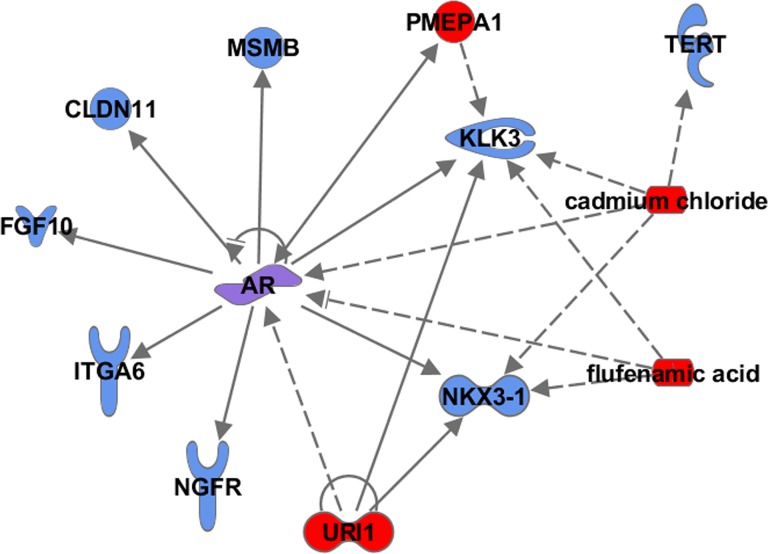
Upstream regulators among genes neighboring SNPs in high LD with prostate cancer risk alleles. The IPA Upstream Regulator tool was used to identify potential upstream regulators based on the statistical significance of genes in the gene list that function downstream of this regulator. The top 5 upstream regulators identified were flufenamic acid, androgen receptor (AR), cadmium chloride, prostate transmembrane protein, androgen induced 1 (PMEPA1), and prefoldin-like chaperone (URI1). Upstream regulators (red); upstream regulator and on the gene list (purple shading); genes from the gene list (blue).

When these 81 focus molecules were queried for alterations to gene expression, copy number, and mutation profiles in TCGA prostate adenocarcinoma dataset (n = 236), we found that more than half of the genes (52 of 81 genes) were altered in at least 5% of the tumors in the dataset, including 5 genes that were altered in >10% of the tumors (S5 Table).We also analyzed the 81 focus molecules for signaling connectivity using the IPA upstream regulator analysis approach and identified 9 significant upstream regulators (Fisher exact test p< 1 x 10^–4^) (S6 Table). The 5 most significant upstream regulators, flufenamic acid, *AR*, cadmium chloride, prostate transmembrane protein, androgen induced 1 (*PMEPA1)*, and URI1, prefoldin-like chaperone (*URI1)* demonstrated connectivity by sharing multiple downstream targets across the gene list (Fig. 4). The most significant upstream regulator was the non-steroidal anti-inflammatory drug, flufenamic acid (FLF). Flufenamic acid functions upstream of the androgen receptor to inhibit *AR* gene expression and through this function has been used as a therapeutic agent for prostate cancer [15]. The androgen receptor was the second most significant upstream regulator with multiple downstream genes on the gene list, namely, fibroblast growth factor 10 (*FGFR10)*, integrin, alpha 6 *(ITGA6)*, *CLDN11*, nerve growth factor receptor *(NGFR)*, *NKX3-1*, *MSMB*, and *KLK3*. The transcription regulator prefoldin-like chaperone (*URI1)* also regulates the expression of the *AR*, as well as a shared downstream gene on the gene list, such as *NKX3-1* [16], a tumor suppressor commonly deleted in prostate tumors. These results indicate that a subset of the genes identified as flanking the prostate cancer index SNPs, or SNPs in high LD with them, may have a key functional role in regulating the genes involved in the androgen receptor-mediated signaling pathway for prostate cancer.

We queried TCGA for alterations in gene expression, copy number, and mutation in the prostate adenocarcinoma dataset (n = 236) using the gene list subset associated with the top five upstream regulators and found that the majority of the genes (6 of 9 genes) were altered in at least 5% of the 236 tumors, with deletion events of *NKX3-1* (frequency: 12.7%) as the most frequent alteration. Other upstream network genes from our gene list that were altered (>5% of the 236) were *ITGA6*, *MSMB*, *CLDN11*, *NGFR*, *FGF10*, *KLK3* (S5 Table).

### Functional Annotation of Prostate Cancer Risk Alleles and SNPs in High LD

We then used the HaploReg tool to identify potential mechanistic functions of non-coding risk alleles by determining whether the SNP of interest is likely to be within exons, promoters, and enhancers of genes at the loci of interest. The HaploReg tool identifies potentially functional SNPs based on regulatory annotations of noncoding sequences based on information from ENCODE [17].

We first focused on the characterization of potential regulatory function for the 86 index SNPs (S7 Table). Two of the 86 SNPs were located in exons, generating missense mutations; one was located in the 3’ UTR region of a gene; 29 were located in intronic regions of genes; seven were located in an evolutionary conserved region predicted to be under functional constraint based on SiPhy (SIte-specific PHYlogenetic) analysis [10]; five contained promoter histone marks; 37 contained enhancer histone marks; 36 were in DNase hypersensitive regions; and 20 were found to have transcription factors bound (based on ChIP-seq) for multiple cell types. These results suggest that many of the 86 prostate cancer risk variants were located in regulatory sequences at transcriptionally active sites. In addition, several risk variants were found to have multiple regulatory features at its locus. One example, rs11568818 at 11q21, is located 182 bp 5’ from the matrix metallopeptidase 7 (*MMP7*) gene. This SNP is within a region with sequence conservation, enhancer histone marks and DNase hypersensitivity in multiple cell types, and, based on ChIP-Seq assays, was found to bind transcription factors, which support the likelihood that this SNP is located in a region with transcriptional regulatory function. In addition, it has been shown by expression quantitative trait loci (eQTL) that transcriptional regulatory function exists at this risk locus for the *MMP7* gene in liver tissue [18].

When the 1,594 SNPs, including the 86 index SNPs and SNPs in high LD with them, were examined using the HaploReg tool, we identified ten SNPs that were located in exons of annotated genes, four located in the 5’UTR, 22 located in the 3’UTR, 15 located in potential promoter regions (<1.5 Kb of the transcriptional start site), 562 located in intronic regions, and 936 located in intergenic regions (S8 Table).

Looking specifically at the promoters (<1.5 Kb from the transcriptional start site) and 5’ and 3’ UTR regions proposed to be involved in transcriptional regulation, we found that 15 SNPs were located in putative promoter regions for 9 different coding genes (*MDM4*, *PIK3C2B*, *MLPH*, *VAMP8*, *NOTCH4*, *MSMB*, *MMP7*, *NGFR*, *VPS53*) and one non-coding gene, cancer susceptibility candidate 8 (*CASC8*). Three SNPs were located in the 5’-UTR of genes (*ZBTB38*, *VPS53*, and *PPP1R14A)*, and 22 SNPs were located in the 3’-UTR of 11 different genes (*MDM4*, *GGCX*, *VAMP8*, *PDLIM5*, *ARMC2*, *SESN1*, *PSORS1C1*, *NKX3-1*, *ZNF652*, *KLK3*, and *LIME1*). SNPs located in the 5’ and 3’-UTR sequences of genes may have an important role in regulating the mechanisms which control gene expression, mRNA stability, and translational efficiency [20].To explore additional regulatory mechanisms for the index risk and linked SNP set, we use the HaploReg tool to identify predicted enhancers and promoters at these loci (S8 Table). The HaploReg tool indicated an overall significant enrichment of enhancers in the stem cell line, H1, with a 12.8 fold (p = 2.9 x 10^–4^) enrichment of enhancer marks over expectation for this SNP set (S9 Table). DNase enrichment analysis indicated a significant enrichment of transcriptionally active sites in the SNP set with a 4.4 fold increase over expected (p = 1.1 x 10^–3^) in a cell line originating from a prostate adenocarcinoma (LNCaP cells), and a 5.8 fold increase over expected (p = 1.8 x 10^–3^) for a prostate epithelial cell line (PrEC). The enrichment of enhancer marks and DNase hypersensitivity marks in stem cells and prostate cancer cells provide strong evidence for the role of prostate cancer risk SNPs and SNPs in high LD with them in regulating gene expression.

For the ten linked SNPs located in exons of characterized genes, five SNPs are synonymous variants, and five are missense variants. One missense variant rs11765552, at 7q21, located in exon 11 (causing L780M) of the *LMTK2* is strongly correlated (r^2^ = 0.99 in Europeans) with the index SNP, rs6465657. We used PolyPhen analysis to predict the functional potential of this amino acid substitution and found that this variant may result in a possibly damaging effect (score 0.761 of 1.00) on protein function of LMTK2. This variant is located very close to the myosin VI binding domain (aa’s 567–773) of LMTK2 [19]. Two of the 5 missense variants are located in the melanophilin (*MLPH)* gene at 2q37. One of the SNPs is an index risk variant (rs2292884) located in exon 10 (causing H347R) and the other SNP (rs2271809) located in exon 11 (E407D) is in high LD (r^2^ = 0.82) with this index risk SNP. We used PolyPhen and PROVEAN analysis to predict the functional potential of these amino acid substitutions, both variants had low predicted protein function disruption. The missense variant at 6p21, rs130067, is an index risk SNP located in exon 7 (E275D) of the coiled-coil alpha-helical rod protein 1 (*CCHCR1)* gene. PolyPhen analysis predicts very low, or benign, potential for protein function disruption (score 0.00 or 1.00). Another missense variant at 20q13, rs8957, is highly correlated (r^2^ = 0.83) with the index risk SNP, rs6062509 and located in exon 6 (E233D) of the solute carrier family 2, *SLC2A4* gene. PolyPhen analysis predicted a very low or benign (score 0.02 of 1.00) potential for protein function disruption for this variant.

## Discussion

We conducted a comprehensive *in silico* functional pathway characterization of the 86 established prostate cancer risk alleles identified to date. To increase our understanding of the biological mechanisms that the index risk variants and near-by genes may be impacting, we took two approaches to identify gene networks that may function in prostate cancer initiation or pathogenesis. One approach was to generate a gene list consisting of annotated genes immediately flanking the index SNP, regardless of distance to the nearest gene. The second approach was to consider the linkage structure and include the neighboring genes of SNPs in high LD (r^2^≥0.8) with the index SNP, within 100 kb. Comparing the two gene lists, there were 58 genes shared between the two lists, 39 genes unique to the list generated by identifying flanking genes and 23 genes unique to the list based on linkage structure. Overall the majority of the genes that were identified were shown to have a biological role in tumorigenesis, with many of the genes having a role in prostate cancer specifically.

With the first approach, using the gene list based on genes flanking the index SNPs, we identified 97 unique genes and 56 of these were cancer-related genes. Seventeen genes were associated with prostate cancer, specifically. With the second approach, evaluating neighboring genes to SNPs in high LD with the risk variants, we identified 78 unique genes. Half of these 78 genes were cancer-related genes, with 14 genes specifically associated with prostate cancer. Not all the genes for the two lists were overlapping, as reflected in the different biological functions of the two top networks for each gene list Table 1 and 2. Both gene lists identified the androgen receptor as a significant upstream regulator, consistent with the known central role of the androgen receptor signaling pathway in prostate cancer [26,27]. Interestingly, the top five significant upstream regulators (androgen, *AR*, *LEF1*, *HH*, and cadmium chloride) based on the flanking gene list, support an additional role for the hedgehog and Wnt/β-catenin signaling pathways pathway. Gowda et al. recently reported that the synergistic inhibition of both the hedgehog and androgen receptor signaling pathways suppressed the growth of castration-resistant prostate cancer, whereas inhibition of either the hedgehog or androgen receptor pathways individually could not achieve similar levels of growth suppression [28], suggesting that both pathways play a critical synergistic role in the biology of prostate cancer. In contrast, the top five significant upstream regulators (flufenamic acid, *AR*, cadmium chloride, *PMEPA1*, and *URI1)* generated, based on the 78 gene list of genes in high LD with the prostate cancer risk variants, primarily function in the androgen receptor signaling pathway and did not identify the hedgehog or Wnt/β-catenin signaling pathways [15,16,29,30]. Overall, these results suggest that the index SNPs are located near genes that may interact and have functional relationships in specific signaling networks. Therefore, assuming that the risk alleles do indeed have regulatory effects on their nearby genes, when considering the functional role of risk alleles, attention to the possibility that multiple variants may function to affect a higher-order network of genes that regulate specific pathways. Additional biological assays will need to be performed to confirm the role of the risk alleles and synergistic interactions between the genes they potentially regulate.

Using the HaploReg tool for the analysis of SNPs and genes in high LD with the index risk variants, we expanded our analysis to include over 1,500 additional SNPs. This expanded analysis pointed to regulatory functions that were not identified with the 86 index SNPs alone, potentially augmenting the list of genes that are associated with the index genetic signals. The majority of these 1,500 SNPs were located in intergenic regions, almost one-third of the linked SNPs were located in intronic regions, a minority of the linked SNPs were located in regulatory domains (promoters and 5’ and 3’ UTRs), and 10 SNPs were located in exons.

When we evaluated the 86 index SNPs for biological features that may impact gene function, rs11568818 at 11q21 displayed the strongest evidence for having transcriptional regulatory function. This variant may directly impact the expression of the *MMP7* gene. It is located in an evolutionary conserved sequence 182 bp 5’ to the *MMP7* gene and contains characteristics of transcriptional regulatory activity, such as histone marks (H3K27Ac) and DNase hypersensitivity. In addition, CHiP assays have identified transcription factors (*TBP*, *FOS*, *JUN*) binding to this region and an eQTL analysis has demonstrated interaction between this SNP and the *MMP7* gene in liver tissue [18]. Allele-specific transactivation of the MMP7 gene by the FOXA2 transcription factor was observed in idiopathic pulmonary fibrosis patients [21]. *MMP7* has been shown to be overexpressed in prostate cancer tissue when compared to normal tissue and was recently demonstrated to be regulated by the ETV1 transcription factor [22]. Regulation by ETV1 is of particular interest because it is a member of the ETS transcription factors. Translocations of the ETS transcription factors, including *ETV1*, occur in half of all prostate cancers resulting in aberrant ETS transcription factor expression which are believed to be an early, potentially initiating event, for prostate cancer [23–25]. Taken together, these data strongly suggest a functional role for rs11568818 in the transcriptional regulation of the *MMP7* gene for prostate cancer. Additional functional studies to examine the potential role of allele-specific transcriptional regulation of *MMP7* by rs11568818 and ETV1 for prostate cancer should be conducted.

Among the SNPs that are in high LD with the prostate cancer index SNPs, our results highlight one of the missense SNPs rs11765552, in high LD (r^2^ = 0.99) with the index risk SNP rs6465657, as being located in the *LMTK2* gene. The *LMTK2* gene encodes for a membrane bound kinase that is involved in intracellular trafficking and endosomal recycling. Puri et al. demonstrated that LMTK2 and myosin VI co-immunoprecipitate and likely function to recycle endosomes [31]. They also showed that overexpression of myosin VI is associated with increased secretion of the prostate surface antigen (PSA) and VEGF. The missense variant rs11765552 is predicted to affect protein structure and function of LMTK2 and potentially binding of LMTK2^L780M^ to myosin VI since this mutation is located near the myosin VI binding domain (aa 567–773) of LMTK2. Therefore, it would be interesting to determine if this missense SNP generates a form of LMTK2 with differential binding capacity for myosin VI, ultimately affecting intracellular trafficking and the endosomal secretory pathway in prostate cancers. *LMTK2* has also been shown to play a critical role in Smad2 mediated TGFβ signal transduction, because LMTK2 stimulates Smad2 binding to kinesin-1 motors and nuclear translocation of Smad2 following TGFβ activation [32].

The HaploReg tool identified two correlated SNPs (rs9643226 and rs1447296) in high LD with the index SNP, rs1447295, located in the putative promoter region of the long non-coding RNA (lncRNA), *CASC8* (cancer susceptibility candidate 8). LncRNAs are non-protein coding RNAs longer than 200 bp and have been implicated in various cellular functions including cancer development [33,34]. Based on regulatory motif prediction models, the two SNPs located in the promoter region of the CASC8 gene could potentially affect transcription factor binding. The regulatory motif prediction models based on the position weight matrices (PWMs) indicate that the reference or alternate allele at the SNP, rs9643226, located 96 bp 5’ from the start site of *CASC8* affects the binding of transcription factors, autoimmune regulator (AIRE) and SRY-related HMG-box (SOX). The alternate allele having increased affinity for AIRE and decreased affinity for SOX compared to the reference allele. For rs1447296, located 947 bp 5’ of *CASC8* start site, regulatory motif prediction indicates binding of transcription factors, STAT and ZNF148, with the alternate allele having greater affinity for STAT and decreased affinity for ZNF148 compared to the reference allele. *CASC8* is located in the gene desert region of 8q24.21, with a paucity of protein coding genes in this 2 Mb region near the *MYC* gene. Multiple cancer-associated variants have been identified in 8q24.21; however, the functional impact of these variants has not been fully characterized. This 8q24 gene desert contains seven lncRNAs, one of which is *CASC8*, also known at *CARLo-1*. In a recent report, Kim et al. evaluated the expression patterns of the seven lncRNAs and found that the expression of one of the seven lncRNAs, *CARLo-5*, is regulated by the TCF4 bound *MYC* enhancer [35]. A similar enhancer-promoter interaction was previously identified between the MYC enhancer at rs6983267 and the promoter of the MYC gene [36,37]. The transcription factor, TCF4, which facilitates this enhancer-promoter interaction has differential binding affinity at the *MYC* enhancer depending on the allele of this established cancer variant rs6983267. Therefore, the rs6983267 allele regulates the expression of CARLo-5 through differential binding to TCF4. The same group also demonstrated that the lncRNA, *CARLo-5*, functions in cell cycle regulation by regulating expression levels of *CDKN1A*. Although the regulation of *CARLo-1* expression and biological function have not been elucidated, it is possible that the two SNPs in the promoter region of *CARLo-1* impact transcription factor binding and expression of *CARLo-1*, and subsequent expression of downstream protein-coding targets, similar to CARLo-5. Interestingly, another correlated SNP, rs1447295, located in a large intron of *CARLo-1* is shown to have a regulatory motif that is predicted to bind to the MYC transcription factor. The position weight matrix modeling show MYC is predicted to have differential affinity for this motif, with the alternate allele having a much stronger binding affinity compared to the reference allele.

Hazelett et al. recently reported comprehensive functional annotation of 77 prostate cancer risk alleles [38]. They evaluated potentially functional SNPs with an r^2^≥0.5 and within a 1 Mb window around the 77 index SNPs. They report on 727 potentially functional SNPs from their analysis, with the majority located in putative enhancer regions. They focused their attention to response elements in promoters or enhancers recognized by known transcription factors having a role in prostate cancer, primarily androgen receptor and in a prostate cancer cell line, LnCaP cells. Our results support their findings that the androgen receptor has a critical role in regulating expression of genes involved in prostate cancer and the GWAS risk markers and their correlated variants may be located in enhancer regions and function to regulate the expression of a subset of these target genes.

Our study demonstrates that the available analytical tools and extensive catalog of non-coding regulatory regions in the genome allow us to explore the potential regulatory function that GWAS risk markers and their highly correlated variants exert on genes in their immediate vicinity and the genes that they regulate may function synergistically in key regulatory pathways.

## Supporting Information

S1 Table Prostate cancer risk variants. (XLSX)

S2 Table List of genes flanking prostate cancer risk variants and distance from index SNP. (XLSX)

S3 Table Gene list of genes flanking prostate cancer risk variants and frequency of alterations in prostate adenocarcinomas included in TCGA dataset (n = 236). (XLSX)

S4 Table Identification of upstream regulators and pathways based on the list of genes flanking the prostate cancer risk variants. (XLSX)

S5 Table Gene list of genes neighboring SNPs in high LD with the prostate cancer risk variants and frequency of alterations in prostate adenocarcinomas included in TCGA dataset (n = 236). (XLSX)

S6 Table Identification of upstream regulators and pathways based on the genes neighboring SNPs in high LD with prostate cancer risk variants. (XLSX)

S7 Table Functional annotation for 86 prostate cancer risk variants. (XLSX)

S8 Table Functional annotation for 86 prostate cancer risk variants and SNPs in high LD (r2>0.80). (XLSX)

S9 Table Enhancer and Dnase enrichment analysis of 86 prostate cancer risk variants. (XLSX)
